# Metabolic Profiling Identifies Changes in the Winter Wheat Grains Following *Fusarium* Treatment at Two Locations in Croatia

**DOI:** 10.3390/plants12040911

**Published:** 2023-02-17

**Authors:** Katarina Sunic, John Charles D’Auria, Bojan Sarkanj, Valentina Spanic

**Affiliations:** 1 Department for Breeding and Genetics of Small Cereal Crops, Agricultural Institute Osijek, Juzno Predgradje 17, 31000 Osijek, Croatia; 2 Department of Molecular Genetics Leibniz, Institute of Plant Genetics and Crop Plant Research (IPK Gatersleben), OT Gatersleben Corrensstraße 3, 06466 Seeland, Germany; 3 Department of Food Technology, University North, Trg dr. Zarka Dolinara 1, 48000 Koprivnica, Croatia

**Keywords:** metabolic profiling, GC-MS, winter wheat, Fusarium head blight, biotic stress

## Abstract

Fusarium head blight (FHB) is one of the most dangerous diseases of winter wheat, resulting in reduced grain yield and quality, and production of mycotoxins by the *Fusarium* fungi. In the present study, changes in the grain metabolomics of winter wheat samples infected with *Fusarium* spp. and corresponding non-infected samples from two locations in Croatia were investigated by GC-MS. A Mann–Whitney test revealed that 24 metabolites detected were significantly separated between *Fusarium*-inoculated and non-infected samples during the variety by treatment interactions. The results confirmed that in grains of six FHB-resistant varieties, ten metabolites were identified as possible resistance-related metabolites. These metabolites included heptadecanoic acid, 9-(Z)-hexadecenoic acid, sophorose, and secolaganin in grains of FHB-resistant varieties at the Osijek location, as well as 2-methylaminomethyltartronic acid, maleamic acid, 4-hydroxyphenylacetonitrile, 1,4-lactonearabinonic acid, secolaganin, and alanine in grains of FHB-resistant varieties at the Tovarnik location. Moreover, on the PCA bi-plot, FHB-susceptible wheat varieties were closer to glycyl proline, decanoic acid, and lactic acid dimer that could have affected other metabolites, and thus, suppressed resistance to FHB. Although defense reactions were genetically conditioned and variety specific, resulting metabolomics changes may give insight into defense-related pathways that could be manipulated to engineer plants with improved resistance to the pathogen.

## 1. Introduction

Cereals such as wheat, barley, oat, rye, and maize account for a considerable portion of the world’s food supply, making them essential in the human diet [1]. Due to the fact that a significant portion of wheat grain yield losses results from different diseases, this crop is constantly threatened [1,2,3]. One of the main threats is Fusarium head blight (FHB), a disease affecting different monocotyledonous plants, and causing substantial damage to wheat. Although caused by numerous species from the genus *Fusarium* [1,4], the most common and predominant causal agents of FHB on wheat are *Fusarium graminearum*, *F. culmorum*, and *F. avenaceum*, although observation of *F. poae* as the cause of disease increased in recent years [4,5]. While different environmental factors can affect *Fusarium* spp. growth and survival [6], the most crucial and frequently associated with the development of FHB are weather conditions at different geographical regions [7]. Except influencing occurrence and incidence of FHB, climatic factors are affecting disease severity as well [8]. Considering different environmental requirements, *Fusarium* species vary depending on their geographical location [9]. Consequently, *F. culmorum*, *F. avenaceum*, and *F. poae* are found to be dominant in the cooler and temperate regions, while *F. graminearum* is common for warmer parts of the world [10]. Although the majority of the *Fusarium* species can be found in nearly all FHB-affected areas, *F. graminearum* appears in a wide range of environmental conditions, making it greatly adapted compared to other species [9]. Since *F. graminearum* is one of the most important *Fusarium* species in Croatia [5,11], during anthesis, when the temperature and humidity are high enough to promote fungal development, it infects the open flowers of host plants [12]. Besides grain yield and quality losses, of particular concern during the *Fusarium* infestation is the accumulation of mycotoxins, which affect all organisms, including humans [13]. However, mycotoxins as secondary metabolites produced by toxigenic fungi of the genus *Fusarium* [4,12] are not the same as the metabolites produced by wheat plants that have a role in FHB defense [14]. Although many sources of moderate FHB resistance have been identified [15], significant progress has been recently made in mapping quantitative trait loci (QTL) for FHB resistance [16] and developing FHB-resistant varieties [17].

Strong evidence suggests that plant metabolism has a direct impact on FHB resistance [15]. Different environmental stresses can affect the synthesis and accumulation of metabolites in cereals, influencing not only the antioxidant activity, but also nutritional quality of grains [18]. Changes in concentration of both secondary and primary metabolites are crucial in defense response of all plant species, including wheat [19]. Processes related to these stress responses affect the growth and development of the plant and include the downregulation of physiological processes such as photosynthesis, respiration, nutrient translocation, and transpiration [20,21]. The predominant metabolites belonging to both primary and secondary metabolism range from amino acids and their derivatives, fatty acids, carbohydrates, amines, and polyamines, as well as terpenoids and phenylpropanoids [22]. Among these groups of metabolites, studies suggested that the phenolic content of the wheat grain was crucial in resistance to *Fusarium* species [23,24]. Except these primary and secondary metabolites, wheat varieties varying in resistance to FHB may produce sets of other metabolites to resist *Fusarium* attack, disease development, and mycotoxin accumulation [25]. Such metabolites could be used as biomarkers or are linked to specific chromosome locations and, as such, could be used in breeding programs [25]. All these changes represent an interesting route for investigating the role of metabolites in wheat–fungus interactions.

Metabolomics has become an indispensable tool in the research of stress interactions in plants [26]. Data obtained during metabolomics analysis offer a deeper insight into the metabolic fluctuations of biological processes during biotic and abiotic stress [27]. This approach can detect different metabolites related to pathogen attack [26], including pathogen-secreted molecules or metabolites produced or mislocalized to enable pathogen growth [28]. Additionally, metabolomics can be effective as a tool to monitor and predict the quality, processing, and safety of raw materials and final products [29]. Beside the prediction of quality traits and major food components such as proteins, lipids, and starch, it can be useful to investigate even less-abundant secondary metabolites [30]. Furthermore, it is an important part of systems biology that could improve our understanding of wheat defense mechanisms as a response to pathogen invasion. Different plant tissues could be used for these kinds of investigations, including wheat grain [31].

As FHB is a dangerous disease resulting in the production of mycotoxins by *Fusarium* fungi in wheat grains, it presents a major problem regarding global food safety, and thus, understanding wheat–fungus interactions is crucial for *Fusarium* disease control. On the opposite of metabolite products accumulated by *Fusarium* spp., plants carry out their own metabolic responses to resist FHB [14,32]. Since most metabolomics studies on cereals are designed to determine the chemical composition of the grains and only few are trying to understand the physiological responses of the plants to internal or external factors, the aim of this study was to detect polar metabolites of the control winter wheat grains and grains under artificial *Fusarium* treatment at two experimental field locations. Therefore, the insight into the possible relationship of certain polar metabolites produced by winter wheat plants with the resistance to *Fusarium* was investigated.

## 2. Results

In the current study, 275 polar metabolites were identified in grains of 25 winter wheat varieties under controlled treatment from two field locations (Osijek and Tovarnik), where no fungicides were used and in grains of corresponding 25 wheat varieties under *Fusarium*-inoculated treatment from the same two locations. A univariate analysis was performed on 275 obtained metabolites (Mann–Whitney test; *p* value < 0.05; N = 200), thus allowing to highlight the metabolites whose amount in grains was significantly modified by *Fusarium* treatment compared to controls (Supplementary Table S1). This approach showed that among the 275 metabolites detected in wheat grain extracts, 24 metabolites (8.73%) from FHB treatment were significantly differentially produced, compared to controlled treatment. Major metabolite changes following *Fusarium* infection in the 25 studied wheat varieties at two locations together are shown in Supplementary Table S1. The agglomerative hierarchical clustering was used to group 25 winter wheat varieties from two locations in controlled and FHB treatment in clusters based on their similarity according to 24 metabolites responses and varietal resistance to FHB. Furthermore, at two locations, different grain metabolites and resistance of winter wheat varieties in two treatments were studied through principal component analysis (PCA) to get an outline of the relationship among investigated parameters.

### 2.1. Tree Diagram of Agglomerative Hierarchical Clustering

**Figure 1:**
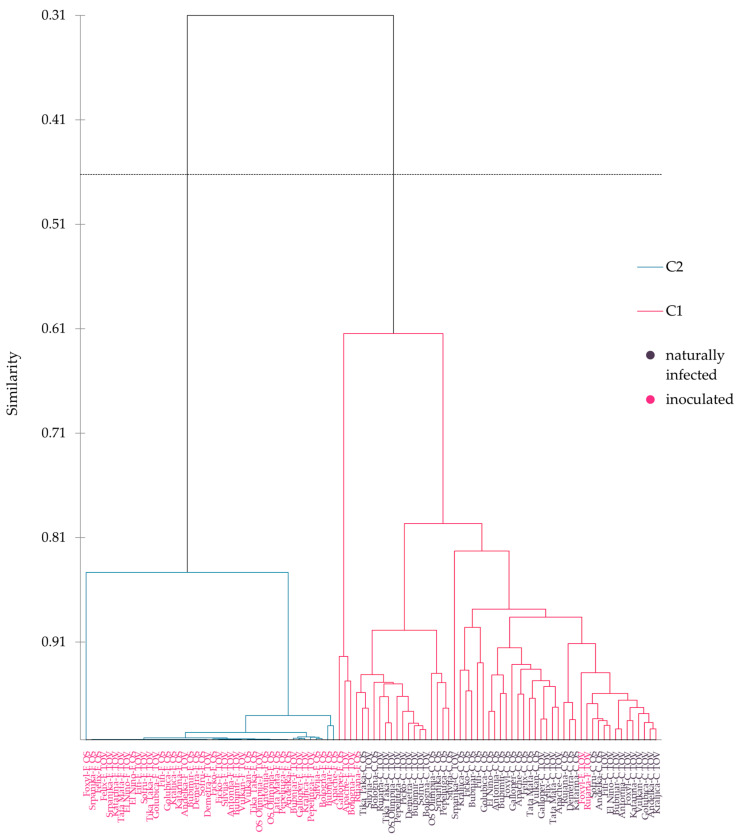
Tree diagram of agglomerative hierarchical clustering of 25 winter wheat varieties based on different grain metabolites and varietal resistance to *Fusarium* in two treatments (naturally infected or controlled treatment-C and inoculated or *Fusarium* treated-F) at both locations. Os—Osijek, Tov—Tovarnik, C1—cluster 1, C2—cluster 2.

In the present study, cluster analysis divided the total of 100 wheat varieties (25 in controlled treatment and 25 corresponding varieties in *Fusarium* treatment at two experimental locations) into two main clusters (Figure 1). The first cluster contained wheat varieties from controlled treatment from both locations, where wheat was under natural infection without usage of fungicides, including six varieties from *Fusarium*-inoculated treatment (Galloper and Rujana from Osijek and Apache, Bologna, Foxyl, and Rujana from Tovarnik). All wheat varieties from natural infection were not showing any symptoms of FHB and as such they could not be scored for FHB severity. Furthermore, six other varieties from *Fusarium*-inoculated treatment had minimal area under the disease progress curve (AUDPC) scores of FHB general resistance (Supplementary Table S2). The second cluster included 44 varieties from treatment with *Fusarium* inoculation from both locations. Furthermore, the second cluster had a sub-cluster at 0.84 similarity and included Foxyl from FHB treatment at Osijek that was different from the rest of the 43 varieties in the same sub-cluster. The highest similarity at Tovarnik was obtained between varieties Felix, Srpanjka, Katarina, Tata Mata, El Nino, Fifi, Sofru, Tika Taka, Golubica, Anđelka, and Demetra, while at Osijek, the highest similarity was obtained between varieties Srpanjka, Felix, El Nino, Fifi, Golubica, Kraljica, Katarina, Bubimir, Demetra, and Sofru. The first cluster showed that under *Fusarium* treatment, varieties such as Galloper at Osijek together with Apache and Bologna at Tovarnik were different from the rest of the varieties under *Fusarium* treatment in the second sub-cluster. The closest distance in the first cluster at Tovarnik was obtained between varieties Sofru and Bologna, Bubnjar and Antonija, and Anđelka and Kraljica (Figure 1).

The average distance from observations to the cluster centroid <1 was obtained in the first cluster under controlled treatment from Tovarnik, including varieties Felix, Anđelka, Kraljica, Bubimir, Apache, El Nino, Galloper, Tata Mata, Sofru, Katarina, Golubica, Fifi, Demetra, and Bologna, and under controlled treatment from Osijek, including varieties Bubimir, Vulkan, Tata Mata, Apache, Felix, and Anđelka, as well as Rujana from FHB treatment at Tovarnik. The highest variability of the observations was observed within the second cluster for Galloper, Pepeljuga, El Nino, Srpanjka, and Felix under FHB treatment at Tovarnik, and for Silvija, Apache, Bubnjar, Foxyl, Felix, and Srpanjka under FHB treatment at Osijek, thus showing the average distance from observations to the cluster centroid >100 (Supplementary Table S3, Supplementary Figures S1 and S2).

### 2.2. Principal Component Analysis of 25 Varieties in Controlled and Fusarium-Inoculated Treatment at Two Locations

**Figure 2:**
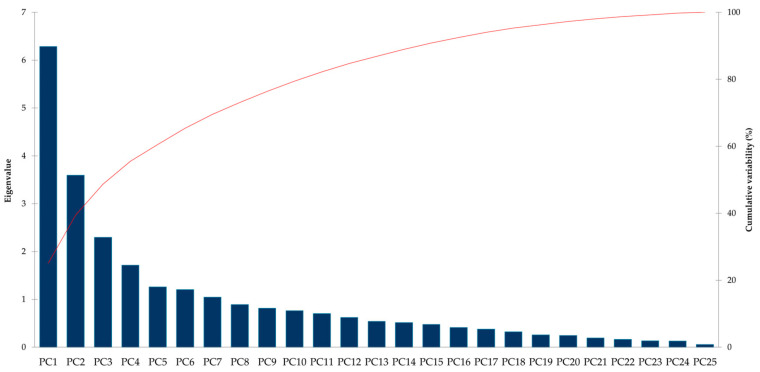
Scree plot showing eigenvalues in response to number of components for the estimated variables of winter wheat varieties at both locations.

The scree plot of the PCA showed that the first ten eigenvalues corresponded to the most percentage of the variance in the dataset. In this study, out of a total of 25 components, 4 had eigenvalues > 1.5 (Figure 2). Four principal components explained approximately 55.54% of the total variability. According to PCA, the variance in the eigenvalues was the greatest for PC1 (6.28), PC2 (3.59), and PC3 (2.29) (Table 1).

PCA showed that PC1 accounted for 25.13% of variation, with sarcosine (10.27), 1,4-lactonearabinonic acid (9.32), pyrrole-2-carboxylic acid (8.91), hydroquinone (8.59), and cembrene (8.45) being the major factors, and thus, showing the largest squared cosine with respect to PC1, while PC2 accounted for 14.38% variation with (3α,5β)-3,21-dihydroxypregnane-11,20-dione (16.91) and sophorose (16.06) as major factors (Table 2). For PC3, major contributors were 2-methylaminomethyltartronic acid (19.47), 9-(Z)-hexadecenoic acid (18.88), and heptadecanoic acid (18.17). The main contributors of PC4 were 2-deoxyguanosine (17.52), tropic acid (14.19), and 2-methylmalic acid (14.18), while the maximum values for various traits in PC5 were varietal resistance to *Fusarium* (25.01) and 2-ethylhexanoic acid (16.97).

**Figure 3:**
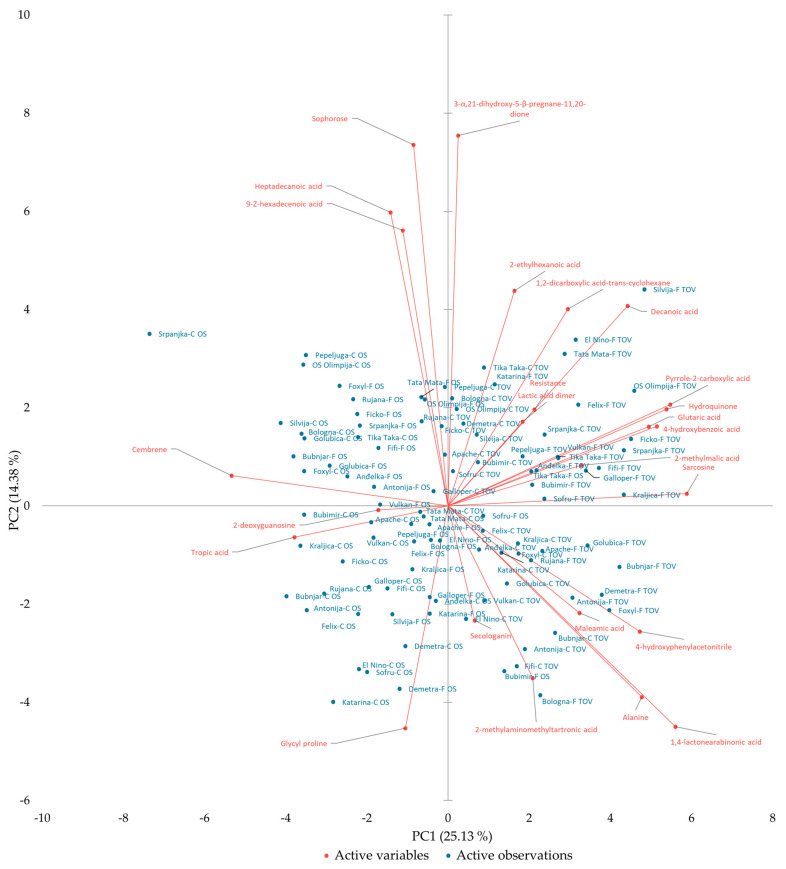
PCA bi-plot of 24 wheat metabolites in the grains and varietal resistance to *Fusarium* of 25 winter wheat varieties under controlled and *Fusarium* treatments at both locations.

On the PCA bi-plot, pyrrole-2-carboxylic acid, 4-hydroxybenzoic acid, glutaric acid, 2-methylmalic acid, and hydroquinone were closely located on the opposite side of tropic acid and 2-deoxyguanosine, indicating that there was direct negative relationship between these metabolites (Figure 3). Furthermore, the metabolites that were negatively correlated included heptadecanoic acid, 9-(Z)-hexadecenoic acid, and sophorose on the upper left quadrant of the PCA bi-plot and secologanin and 2-methylaminomethyltartronic acid on the lower right quadrant of the PCA bi-plot. Furthermore, the closest to varietal resistance to *Fusarium* was lactic acid dimer. Grouping of the metabolites on the same side of the bi-plot indicated non-significant difference between them. The distance of each variable with respect to PC1 showed the contribution of each variable in the variation of germplasm. PC analysis showed grouping of both control and *Fusarium*-treated varieties from different locations on the opposite sides, with varieties from Tovarnik on the right and varieties from Osijek on the left side of bi-plot (Figure 3). FHB-susceptible varieties El Nino, Tata Mata, Felix, and Srpanjka under *Fusarium* treatment at Tovarnik grouped together on the upper right quadrant of the PCA bi-plot, while resistant varieties such as Apache, Rujana, Bubnjar, and Foxyl under *Fusarium* treatment at Tovarnik were distributed further away and grouped together on the lower right quadrant of the bi-plot. Similar results were obtained for Osijek. Varieties resistant to FHB under *Fusarium* treatment grouped together on the upper left quadrant of the bi-plot, while varieties susceptible to *Fusarium* under the *Fusarium* treatment grouped on the opposite side on the lower left quadrant. However, variety susceptible to FHB under *Fusarium* treatment, Srpanjka, was situated on the upper left quadrant further away from other susceptible varieties (Figure 3). The correlation coefficient between any two traits is approximated by the cosine of the angle between their vectors in the plot of the first two PCs and the most prominent relations were between lactic acid dimer and varietal resistance to *Fusarium*, between hydroquinone and pyrrole-2-carboxylic acid, between 4-hydroxybenzoic acid and glutaric acid, as well as between alanine and 1,4-lactonearabinonic acid, as indicated by the small obtuse angles between their vectors (Figure 3).

**Figure 4:**
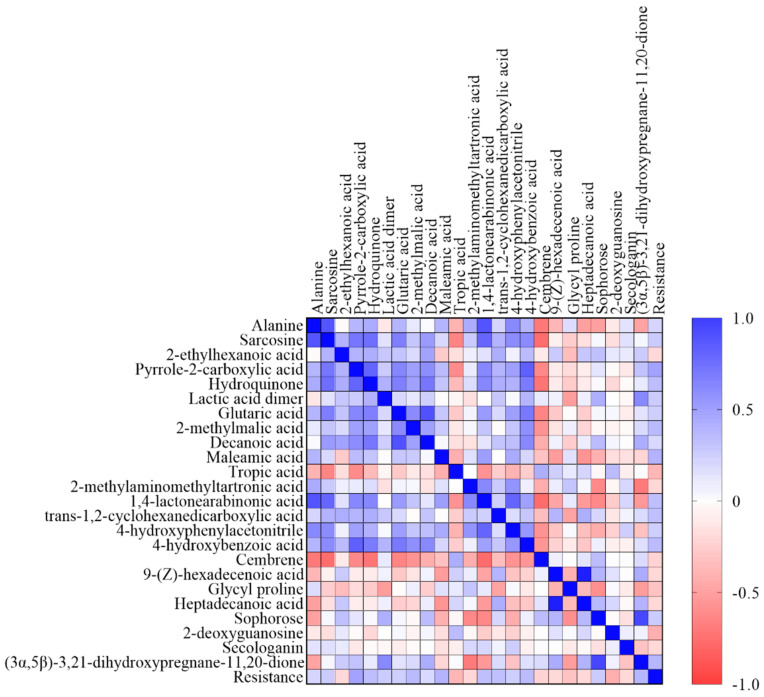
Graphical Spearman correlation matrix of 24 wheat metabolites in grains and varietal resistance to *Fusarium*. Spearman correlation r values were determined using GraphPad Prism 9.4.1. Colors are added for better visualization. The colors span from dark blue to dark red, where dark blue denotes an r value of 1 and dark red indicates an r value of −1.

Spearman correlation coefficient showed that among 24 metabolites, all of them correlated with at least one significant correlation with each other (Figure 4, Supplementary Table S4). The most distinguished positive correlations were between amino acids alanine and sarcosine, as well as between heptadecanoic acid and 9-(Z)-hexadecenoic acid, while the most prominent negative correlations were obtained between cembrene and alanine, cembrene and sarcosine, cembrene and hydroquinone, as well as between cembrene and 1,4-lactonearabinonic acid.

## 3. Discussion

Metabolites are the end products of cellular metabolism with fluctuations in their concentrations as a result of the individual’s phenotype to genetic or environmental changes [33]. Results of the Mann–Whitney U test in the current study revealed that exposure to FHB significantly affected 24 wheat grain metabolites at two locations together among 275 detected polar metabolites. Previously, metabolic profiling of barley spikelets inoculated with *F. graminearum* identified hundreds of metabolites [34]. It was of interest in the current research to obtain metabolite profiles of wheat grains and the final products of plant development, as well as to determine the potential association of resistance related (RR) metabolites in the wheat grains with the FHB resistance or susceptibility during maturity of wheat plants. The metabolites detected to significantly vary between treatments belonged to diverse functional groups including amino acids and amines (alanine, sarcosine, glycyl proline), saturated fatty acids (2-ethylhexanoic acid, heptadecanoic acid, decanoic acid) and unsaturated fatty acids (9-(Z)-hexadecenoic acid), polyphenols and their derivatives (hydroquinone, 4-hydroxybenzoic acid), nucleotides (2-deoxyguanosine), terpenoids (secologanin, cembrene), benzyl cyanides (4-hydroxyphenylacetonitrile), small organic (carboxylic) acids (pyrrole-2-carboxylic acid, tropic acid, glutaric acid, 2-methylmalic acid, maleamic acid, lactic acid dimer, 2-methylaminomethyltartronic acid, 1,4-lactonearabinonic acid, trans-1,2-cyclohexanedicarboxylic acid), hydroxysteroids ((3α,5β)-3,21-dihydroxypregnane-11,20-dione), as well as the carbohydrates (sophorose).

### 3.1. FHB-Resistant Wheat Varieties and Their Clustering

The most important aim in wheat breeding for FHB resistance is that resistant wheat varieties should develop low symptom severity when testing is performed under *Fusarium* artificial inoculations [35]. Under both locations studied in the current experiments, there were few wheat varieties with low AUDPC for FHB severity, meaning they have a higher general resistance to FHB. The tree diagram obtained according to agglomerative hierarchical clustering differentiated wheat varieties into two distinct clusters. Three winter wheat varieties were clustered in one clade, namely Galloper from FHB treatment at Osijek, as well as Apache and Bologna from FHB treatment at Tovarnik. These varieties differentiated from the rest of varieties in the first cluster, which contained almost all varieties from controlled treatment without symptoms of FHB infection. Previously, it was concluded that varieties with a high level of resistance to FHB were visually symptomless on the spikelets [36]. Three other varieties from FHB treatment, namely Foxyl and Rujana at Tovarnik and Rujana from FHB treatment at Osijek, also clustered together in the same cluster, similar to three other FHB-resistant varieties. According to these observations in tree diagram, it can be hypothesized that those six varieties possessed FHB resistance. Some of these varieties were already characterized as FHB resistant in previous research [37], and according to our previous field experiments for breeding purposes, but those data were not publicized. Plants are usually screened for FHB resistance by spray inoculation, as it is the case in the current research. However, because of the generally polygenic nature of resistance and high level of genotype-by-environment interactions FHB is particularly challenging disease when it comes to resistance screening [38]. Some other varieties, such as Bologna, Kraljica, and Galloper from Tovarnik and Galloper and Foxyl from Osijek, also showed FHB resistance, although they were not clustered with control plants. Furthermore, varieties and 24 metabolites were subjected to PC analysis. By reducing the number of dimensions in the data, PCA enables visualization while preserving as much of the original data’s information as possible [39]. Because of that reason, PCA is a powerful tool which enables identification of data patterns, while highlighting similarities or differences present in a dataset [40].

### 3.2. Polar Metabolites Related to Wheat Varieties Possessing FHB Resistance

#### 3.2.1. Saturated and Unsaturated Fatty Acids, Carbohydrates, Terpenoids, and Organic Acids

Varieties Rujana and Foxyl, declared as resistant varieties according to their AUDPC values for general resistance, decreased heptadecanoic acid and 9-(Z)-hexadecenoic acid and increased sophorose under *Fusarium* treatment compared to controls at Osijek. These metabolites were near Rujana and Foxyl on the PCA bi-plot and could potentially affect their resistance to *Fusarium* spp. In support of that is the fact that heptadecanoic acid (C 17: 0, margarinic or margaric acid) is a saturated fatty acid and that 9-(Z)-hexadecenoic (C 16: 1, palmitoleic acid) is one of the main unsaturated fatty acids of wheat [41,42], where previous research reported that approximately 40 identified metabolites associated with fatty acid metabolic pathways may potentially affect cereal resistance against *F. graminearum* [43]. Furthermore, these metabolites from fatty acid metabolic pathways could have role in basal immunity and gene-mediated resistance in plants [44], but could also be involved in the breaking down of products such as oxylipins [45]. It is known that plants produce oxylipins for different purposes [46]. Some studies reported that oxylipins function as metabolites acting directly against various pathogens (such as *Fusarium* spp.), but also as attractors of biocontrol agents [47,48]. Additionally, heptadecanoic acid and 9-(Z)-hexadecenoic acid are part of wheat lipids that contribute to wheat grain quality.

Sophorose, a dimer of glucose that belongs to carbohydrates, was located on the PCA bi-plot near heptadecanoic acid and 9-(Z)-hexadecenoic acid. Increasing carbohydrates might indicate changes of the cell wall structures upon pathogen attack. This was already reported before, where it was concluded that an increase in carbohydrate concentrations might be a fortification of the cell wall barrier in order to prevent *F. graminearum* penetration [49]. It is also known that carbohydrates and their derivatives play a role in cell signaling by enhancing the expression of different defense-related genes, as well as in membrane biogenesis [21,25]. Sophorose was also one of the major factors that contributed to PC2, while 9-(Z)-hexadecenoic acid and heptadecanoic acid were one of the main contributors to PC3. Furthermore, heptadecanoic acid and 9-(Z)-hexadecenoic acid were in proximity to one another on the same quadrant of the biplot and all shared vectors in the same direction. Although Spearman correlation coefficient between heptadecanoic acid and 9-(Z)-hexadecenoic acid was highly significant in correlation matrix, both of these acids were not in significant correlation to varietal resistance to *Fusarium*. However, sophorose showed significant positive correlation with varietal resistance to FHB. In previous research [50], a negative significant correlation was found between heptadecanoic acid and *Fusarium* wilt incidence, where authors concluded that a positive r value may indicate that fatty acids had stimulatory effects on the growth and sporulation of cottonseed fungi, while a negative r value could be attributed to inhibitory activities of fatty acids. Thus, it can be concluded that in the current research, sophorose directly influenced varietal resistance to *Fusarium*, while heptadecanoic acid and 9-(Z)-hexadecenoic acid through lipid metabolism indirectly influenced resistance.

On the opposite side of heptadecanoic acid, 9-(Z)-hexadecenoic acid, and sophorose on the PC bi-plot were 2-methylaminomethyltartronic (tartronic) acid and secologanin. 2-methylaminomethyltartronic acid was decreased in Rujana and Foxyl at Osijek. However, variety Bologna at Tovarnik under *Fusarium* treatment, located near 2-methylaminomethyltartronic acid on the bi-plot, increased this acid. In addition to the PCA display, the correlation matrix confirmed significant negative correlation between sophorose and 2-methylaminomethyltartronic acid. 2-methylaminomethyltartronic acid is a dicarboxylic (organic) acid related to malonic acid [51], where the ionized form of malonic acid, malonate, is found to accumulate in plants as a response to abiotic stress, although not as a primary defense metabolite [52]. Considering accumulation of the malonate under certain stress conditions, it can be hypothesized that 2-methylaminometyltartronic acid also accumulates following stress induced by a pathogen attack that will enable FHB resistance in some varieties.

Secologanin was located near 2-methylaminomethyltartronic acid in our analysis. Secologanin is a secoiridoid glucoside and a pivotal terpenoid intermediate in the biosynthesis of indole alkaloids. Secologanin was increased in FHB-resistant variety Foxyl under FHB-inoculated treatment compared to controls at Osijek, while another FHB-resistant variety, Rujana, exhibited slightly decreased levels. Previously, it was reported that secologanin accumulates in barley lines that were more resistant to FHB [53]. On the PCA, very close to secologanin, the FHB-resistant variety Galloper under *Fusarium*-inoculated treatment in Osijek was present. However, although secologanin in the correlation matrix was not significantly correlated to most of the metabolites, there was a negative correlation with (3α,5β)-3,21-dihydroxypregnane-11,20-dione. Since secologanin was increased in varieties Foxyl and Galloper, which are also considered FHB resistant (according to low AUDPC values), it can be assumed that secologanin plays a role in resistance to FHB. Thus, the evidence suggests that metabolites contributing to FHB resistance in above-mentioned varieties (Foxyl, Rujana, Bologna, and Galloper) were saturated and unsaturated fatty acids together with carbohydrate sophorose, terpenoid secologanin, as well as organic acid 2-methylaminomethyltartronic acid.

#### 3.2.2. Amino Acids, Small Organic (Carboxylic) Acids, and Benzyl Cyanides

Varieties Kraljica and Galloper, also considered as FHB-resistant varieties according to their AUDPC values for general resistance, at Tovarnik under *Fusarium* treatment were located near metabolites sarcosine and 2-methylmalic acid on the PCA bi-plot. Sarcosine is an amino acid and intermediate of glycine betaine, a metabolite known for its accumulation in stress conditions in plants. The decline in sarcosine levels relates to the biosynthesis of glycine betaine and plant defense against various kinds of stresses [54]. However, in the current research, varieties Kraljica and Galloper exhibited an increase in sarcosine levels following *Fusarium* treatment, compared to control plants. Nevertheless, amino acids represent building blocks for several other biosynthetic pathways and play pivotal roles during signaling processes as well as in plant stress response [55], besides their fundamental role in synthesis of peptides and proteins [12]. Sarcosine has a role as methyl donor for antioxidative metabolites in plant stress metabolism [56]. In the correlation matrix, sarcosine was significantly positively related to other amino acids, fatty acids, organic acids, phenols and derivatives, and benzyl cyanides, and negatively related to metabolites that negatively correlated with varietal resistance to FHB, namely terpenoids and organic acids (cembrene and tropic acid). Sarcosine was also one of the main contributors to PC1.

Another metabolite closely located to varieties Kraljica and Galloper on the PCA bi-plot was 2-methylmalic acid (malic acid derivative), which increased in grains of both of those varieties after FHB treatment compared to controls. Malic acid is a small organic acid that participates in a plethora of different metabolic pathways. Certain enzymes are directly involved in malate metabolism and one of these enzymes is NADP-malic enzyme. One of these roles of NADP-malic enzyme could be in plant defense against pathogen attack by catalyzing production of NADPH [57]. NADPH is needed for production of reactive oxygen species [58], particularly H_2_O_2_, which is crucial as a plant regulatory molecule [59], and is involved in reactions such as lignification [60]. It is also assumed that long-term stress exposure results in the increase of 2-methylmalic acid in plants [61], while the addition of the exogenous malic acid enhanced the activity of antioxidant enzymes in *Pinus massoniana* [62]. Fusarium wilt infection of watermelon also increased levels of malic acid, indicating that malic acid acts as a defense-signaling molecule [63]. In the correlation matrix, 2-methylmalic acid was significantly positively correlated with amino acids, organic acids, phenols and derivatives, fatty acids, and varietal resistance to FHB, and negatively correlated with terpenoids (cembrene) and fatty acids (9-(Z)-hexadecenoic acid). Since 2-methylmalic acid correlated positively with phenolic compounds such as hydroquinone or 4-hydroxybenzoic acid, it could be hypothesized that 2-methylmalic acid plays a role in plant defense against *Fusarium* infection.

Other FHB-resistant varieties according to their AUDPC values for general resistance at Tovarnik under *Fusarium* treatment, i.e., Foxyl, Apache, and Rujana, also grouped together on the PCA bi-plot. These varieties were closely located to maleamic acid, alanine, 4-hydroxyphenylacetonitrile, as well as 1,4-lactonearabinonic acid. In FHB-resistant varieties Foxyl, Apache, and Rujana, maleamic acid was increased under FHB treatment compared to controls. Previous research reported on the decrease of maleamic acid under drought stress [64]. However, the authors found no evidence that this metabolite plays a specific role in plant resistance to stress conditions. Furthermore, this acid was one of the metabolites whose content was significantly changed in seedlings of blackgram under salinity stress [65]. Although its role in plant stress response is not yet fully understood, it is known that some organic acids play an important role in plant responses to biotic stress [66], e.g., reduced levels of some small organic acids can enhance plant host innate immunity towards fungal pathogen by affecting expression of signaling and structural proteins [67]. In addition to roles of small organic acids in varietal resistance, in the current research in the correlation matrix, maleamic acid was positively correlated with amino acids, organic acids, phenols and derivatives, benzyl cyanides, and varietal resistance to FHB, while negatively correlated with fatty acids, organic acids, terpenoids, and sugars.

Alanine, a non-essential amino acid, increased in FHB-resistant varieties Rujana and Apache in *Fusarium*-treated plants compared to controls. This is in accordance with several studies that reported the accumulation of alanine upon pathogen attack, indicating its role as a possible marker of pathogen infection as well as a protective role against biotic stress [68,69,70]. However, FHB-resistant variety Foxyl at Tovarnik decreased this amino acid in FHB treatment, compared to controls, although this decrease was negligible. According to the correlation matrix for this study, alanine significantly positively correlated with other amino acids, organic acids, phenols and derivatives, and benzyl cyanides, and negatively correlated with organic acids, fatty acids, terpenoids, hydroxysteroids, and carbohydrates. Since the exact reason of stress-induced accumulation of alanine is still unclear and considering the fact that change in alanine concentration could be due to hypoxia [71] and other unfavorable conditions such as low temperatures [72], more research should be focused on this topic. However, since this amino acid also correlated positively with phenolic compounds, it can be hypothesized that its elevated concentrations of alanine could have a role in plant defense against fungal pathogens.

1,4-lactonearabinonic acid increased in varieties Foxyl, Apache, and Rujana at Tovarnik under *Fusarium* treatment compared to controls. While for alanine there are numerous scientific evidences on its role in plant stress, for 1,4-lactonearabinonic acid (derivative of arabinonic acid), there are not much data. Arabinonic acid belongs to the class of organic compounds known as sugar acids derivatives. This acid correlated significantly positively with amino acids, organic acids, phenols and derivatives, benzyl cyanides, and varietal resistance to FHB and correlated negatively with organic acids (tropic acid), terpenoids, fatty acids, carbohydrates, nucleotides, and hydroxysteroids.

4-hydroxyphenylacetonitrile was elevated in FHB treatment compared to controls in all three mentioned varieties (Foxyl, Apache, and Rujana) at Tovarnik. Previous studies reported 4-hydroxyphenylacetonitrile as an intermediate in the biosynthesis of dhurrin and cyanogenic glycoside, which is found in many *Poaceae* species, which plays a role as a plant defense compound [73].

From Figure 1, Foxyl, Apache, and Rujana under FHB treatment from Tovarnik were located in the first cluster with asymptomatic controlled treatment and three other FHB-resistant varieties from FHB treatment, thus implicating their FHB resistance. We hypothesize that the main contribution to resistance of these varieties can be attributed to small organic (carboxylic) acids, amino acids, and benzyl cyanides. All wheat varieties (Rujana and Galloper at Osijek, and Apache, Bologna, Foxyl, and Rujana from Tovarnik) that were related via the aforementioned polar metabolites were artificially *Fusarium* inoculated and belonged to the first cluster, where the majority of wheat varieties were in controlled treatment and had no visible FHB infection on the wheat heads. Additionally, Foxyl from *Fusarium*-treated plants at Osijek was located under second cluster completely differing from the rest of varieties at the same cluster. The other varieties mentioned, such as Kraljica and Galloper under FHB treatment at Tovarnik, possessed FHB resistance but were differentiated from other resistant varieties on the tree diagram according to different mode of action of polar metabolites where main contributors of resistance were sarcosine and 2-methylmalic acid. Nevertheless, we can assume that FHB resistance and defense reactions of the varieties mentioned above are genetically conditioned and variety specific.

### 3.3. Potential Influence of Polar Metabolites on FHB-Susceptible Varieties

In the second cluster of the tree diagram, varieties under *Fusarium* treatment from both experimental locations were located, except six varieties from FHB treatment placed in the first cluster. The highest similarity in the second cluster was obtained between varieties Srpanjka, Felix, El Nino, Fifi, Golubica, Kraljica, Katarina, Bubimir, Demetra, and Sofru at Osijek, while at Tovarnik, the highest similarity was obtained between varieties Felix, Srpanjka, Katarina, Tata Mata, El Nino, Fifi, Sofru, Tika Taka, Golubica, Anđelka, and Demetra. Almost all of the above-mentioned varieties were declared as FHB susceptible or at least moderately susceptible according to their increased AUDPC values for general resistance. Only varieties from the second cluster that were not classified as susceptible or moderately susceptible according to their AUDPC values for general resistance were Kraljica at Osijek, variety Tika Taka at Tovarnik, and Foxyl at Osijek. Higher AUDPC values for general resistance were recorded at experimental Tovarnik, thus indicating more pronounced infection with *Fusarium* species. Therefore, higher disease severity at Tovarnik was strongly driven by microclimate conditions, more specifically by temperature and humidity, both of which were elevated at Tovarnik in comparison to Osijek. Previous studies reported that the coincidence of the higher temperatures and wet conditions during period of anthesis enhanced the infection with *Fusarium* species [9,74,75]. The highest variability of polar metabolites was observed within second cluster for Galloper, Pepeljuga, El Nino, Srpanjka, and Felix under FHB treatment at Tovarnik, and for Silvija, Apache, Bubnjar, Foxyl, Felix, and Srpanjka under FHB treatment at Osijek, thus showing a great proportion of influence of *Fusarium* infection on polar metabolites.

El Nino and Felix were declared as susceptible varieties to FHB, due to having more than 200 AUDPC units. El Nino decreased glycyl proline under *Fusarium*-inoculated treatment in Osijek, compared to controlled treatment, while Felix increased it but still was on the same quadrant with El Nino on the PCA bi-plot. At the Tovarnik experimental location, both varieties decreased this metabolite under FHB treatment compared to control plants. It is important to note that glycyl proline is related to major organic osmolytes glycine and proline with a previous report stating that proline levels during pathogen infection depended on the physiology of the host–pathogen interactions [76]. Glycyl proline was significantly negatively correlated with fatty acids, phenols and derivatives, organic acids, hydroxysteroids, carbohydrates, and varietal resistance to FHB. This also confirmed the previous observation concerning the influence of fatty acids (9-(Z)-hexadecenoic and heptadecanoic acid) and carbohydrates (sophorose) on resistance to FHB. A significant positive correlation of listed polar metabolites was only obtained with 2-methylaminomethyltartronic acid that belongs to small organic (carboxylic) acids.

Furthermore, varieties susceptible to FHB, El Nino and Tata Mata, increased decanoic acid in *Fusarium*-inoculated plants compared to controls at Tovarnik. As decanoic acid is a saturated fatty acid, it plays vital roles as a precursor of sphingolipids, surface waxes, and cutin, and is involved in protein acylation [77]. Furthermore, changes in the levels of saturated fatty acids, including decanoic acid, was observed in *Catharanthus roseus* plants under cadmium stress where its content also increased [78]. In previous research, similarly, saturated fatty acid and dodecanoic acid also increased in switchgrass under conditions of water deficit [79]. However, at the Osijek experimental location, varieties El Nino and Tata Mata decreased the decanoic acid under *Fusarium* treatment compared to control plants. In the correlation matrix, decanoic acid correlated most prominently with glutaric acid, while other significant positive correlations were obtained with amino acids, organic acids, phenols and derivatives, benzyl cyanides, carbohydrates, and hydroxysteroids. Significant negative correlation was obtained with terpenoids (cembrene). Besides decanoic acid, El Nino and Tata Mata increased lactic acid dimer at both locations under FHB treatment. Plant–pathogen interactions can lead to internal hypoxia of both host and pathogen [80,81]. As the infection proceeds, photosynthetic rates are reduced, consequently resulting in reduced oxygen levels and further production of reactive oxygen species (ROS) at the site of infection. This will result in the reduction of oxygen in plants undergoing pathogen attack [82]. Since one of the main products during hypoxia or anoxia conditions in the soil is lactic acid, it could be hypothesized that during hypoxic conditions induced by pathogen infection, in this case FHB, increased production of lactic acid in FHB-susceptible varieties occurs. Although the relation between lactic acid and varietal resistance to FHB on the PCA bi-plot was prominent, the correlation matrix showed that the lactic acid dimer did not correlate significantly with varietal resistance to FHB. However, other significantly positive correlations obtained for lactic acid dimer were with organic acids, fatty acids, phenols and derivatives, carbohydrates, and hydroxysteroids, while negative correlation was obtained with amino acids and amines (glycyl proline).

Most of the metabolites mentioned correlated negatively with the terpenoid cembrene. Cembrenoids are metabolites with inhibitory effects on plant growth and fungal spore germination commonly found in plants [83,84]. Furthermore, [85] reported *Fusarium* infection upregulated terpenoid biosynthesis, and consequently, activated early plant responses to the infection. We then suggest that in the current research metabolites belonging to amino acids and amines, saturated fatty acids, small organic (carboxylic) acid, and terpenoids could have affected other metabolites and thus suppressed resistance of the above-mentioned varieties to FHB. In general, as most of metabolites significantly negatively or positively correlated herein, it can be hypothesized that all metabolites functioned in specific connections, resulting in suppression or enhancement of each other.

## 4. Materials and Methods

### 4.1. Plant Material and Field Experiments

The entire field experiment was conducted in vegetative season 2019/2020 at Osijek (45°32′ N, 18°44′ E) and Tovarnik (45°10′ N, 19°09′ E), Croatia. The soil types at these two regions are the major soil types used for crop production in continental Croatia, eutric cambisol and black soil chernozem, respectively. According to data of the Croatian meteorological and hydrological service, the precipitation during the growing season was 408.6 mm in Osijek and 448.3 mm in Tovarnik, and the average temperature was 11.1 °C in Osijek and 11.7 °C in Tovarnik. The amount of precipitation during May, at the flowering stage, when the plants are the most vulnerable to FHB, was 53.3 mm in Osijek and 58.8 mm in Tovarnik, while the average temperature was 15.3 °C in Osijek and 15.6 °C in Tovarnik (Supplementary Figures S3 and S4). The total of 21 winter wheat (*Triticum aestivum* L.) varieties (Bubnjar, Antonija, Galloper, Tika Taka, Kraljica, Tata Mata, Demetra, Rujana, Ficko, Katarina, Pepeljuga, Silvija, El Nino, Felix, Vulkan, Anđelka, Fifi, Srpanjka, OS Olimpija, Golubica, and Bubimir) used in this research originated from the Agricultural Institute Osijek, with four wheat varieties from foreign institutions (Foxyl, Sofru, Apache, and Bologna). Those 25 varieties were sown with a plot sowing machine (Hege 80, Wintersteiger) in October of 2019 in 7.56 m^2^ plots at the experimental field of the Agricultural Institute Osijek at Osijek and at the experimental field of Agro-Tovarnik at Tovarnik. The field experiment was set up in randomized complete block design where two replications were subjected to artificial *Fusarium* treatment and two replications were under natural infection treatment. The seed density was 330 seed m^−2^ for all wheat varieties. Except fungicide application, which was excluded in these experiments, the agro-technical practices utilized were usual for commercial wheat cultivation in Croatia. In July of 2020, experimental plots were harvested with a Wintersteiger cereal plot combine-harvester and grains were stored until further analyses.

### 4.2. Inoculum Preparation and Inoculation Procedure

The *Fusarium* species used in this experiment were *F. graminearum* (PIO 31), isolated from the winter wheat collected in the eastern part of Croatia, and *F. culmorum* (IFA 104), obtained from IFA-Tulln, Austria. Conidial inoculum of *Fusarium* spp. was produced by a mixture of wheat and oat grains (3:1 by volume). Conidial concentrations of both fungi were determined using a hemocytometer (Bürker-Türk, Hecht Assistent, Sondheim vor der Rhön, Germany) and were set to 1 × 10^5^ mL^−1^. The 100 mL of inoculum was sprayed with sprayers on an area of m^2^ when 50% of the plants inside each plot were at the flowering stage (Zadok’s scale 65) [86]. One treatment was grown according to standard agronomical practice with no usage of fungicide and without misting treatment, while another treatment was subjected to two inoculation events two days apart using a tractor-back (Osijek) and hand sprayer (Tovarnik). Misting was provided by water spraying with sprinklers on several occasions after inoculations.

### 4.3. Disease Severity Asssessment

General resistance (disease severity) to FHB of wheat varieties was evaluated according to a linear scale (0–100%) on days 10, 14, 18, 22, and 26 after inoculation by assessing the whole plot area that consisted of 4400–4600 plants, after which *AUDPC* was calculated according to the following formula:

$$\mathrm{AUDPC}=\overset{n}{\underset{i=1}{\sum }}\left\{\left[\frac{Yi+Yi-1}{2}\right]*\left(Xi-Xi-1\right)\right\}$$

where *Yi* is percentage of visibly infected spikelets (*Yi*/100) at the *i*th observation, *Xi* is day of the *i*th observation, and *n* is total number of observations (Supplementary Table S2).

### 4.4. Metabolite Profiling

Polar metabolites were extracted from 15 mg of deep-frozen, homogenized plant material using a previously described liquid–liquid extraction protocol [87,88,89,90]. Extraction proceeded by adding 1 mL chilled extraction buffer (2.5:1:1 *v*/*v* MeOH/CHCl3/H_2_O) containing 1 μL of a 2 mg/mL stock solution of 13C-sorbitol, and D4-Alanine to the flash frozen and pulverized tissue. Following 15 min incubation at 4 °C, 0.400 mL H_2_O was added and the extraction was split into three batches and aliquots of 50 μL of polar phase were sampled. The dried extracts were in-line derivatized directly prior to injection [91] using a Gerstel MPS2-XL autosampler (Gerstel, Mühlheim/Ruhr, Germany) and analyzed in split mode (1:3) using a LECO Pegasus HT time-of-flight mass spectrometer (LECO, St. Joseph, MI, USA) connected with an Agilent 7890 gas chromatograph (Agilent, Santa Clara, CA, USA). Sample identification of known and unknown features occurred using the LECO ChromaTOF software package while using the Golm Metabolome Database (GMD). Peak intensities were determined using the R package TargetSearch [92] and normalized regarding to sample weight, internal standards, and measuring day/detector response. Metabolites showing >5% missing values among the samples were excluded from the analysis.

### 4.5. Statistical Analysis

Statistical analysis of the normalized, outlier-corrected data was performed using the Statistica 12.0 software (StatSoft Inc., Tulsa, OK, USA). Metabolomics data were analyzed using univariate (Mann–Whitney U test) analysis to prove if there were any significant differences between two treatments (control, i.e., naturally infected, and artificially infected, i.e., inoculated). Further data processing and multivariate analysis, including agglomerative hierarchical clustering and principal component analysis, were performed using Addinsoft XLSTAT (New York, NY, USA) [93], while Spearman correlation r values were determined using GraphPad Prism 9.4.1 [94].

## 5. Conclusions

Winter wheat has developed different mechanisms to overcome biotic stress induced by pathogen attack. Inoculations with *Fusarium* spp. triggered a complex network of resistance-related mechanisms and one of the ways to understand them could be metabolic profiling. In the present study, metabolic profiling of 25 winter wheat varieties at two different experimental locations revealed that out of 275 grain polar metabolites, 24 metabolites were significantly separated during the variety by treatment interaction. The metabolites that varied between treatments belonged to diverse functional groups, such as amino acids and amines, saturated and unsaturated fatty acids, polyphenols and their derivatives, nucleotides, terpenoids, benzyl cyanides, small organic (carboxylic) acids, hydroxysteroids, and carbohydrates. Principal component analysis of metabolite profiles revealed a separation of the wheat varieties from the majority of FHB treatment with the varieties from control, while ten metabolites in the grains of FHB-resistant varieties were identified as possible resistance-related (RR) metabolites. Nevertheless, we suggest that defense reactions were variety specific where cross-talk of metabolites suppressed or enhanced each other. Therefore, metabolomics research based on mass spectrometric techniques is an important part of systems biology that could improve our understanding of plant defense mechanisms as a response to *Fusarium* pathogen invasion.

## Figures and Tables

**Table 1:** Principal component analysis of 24 different wheat metabolites and resistance to *Fusarium* at both locations.

	PC1	PC2	PC3	PC4	PC5	PC6	PC7	PC8	PC9	PC10
Eigenvalue	6.282	3.594	2.294	1.715	1.257	1.204	1.045	0.889	0.816	0.764
Variability (%)	25.129	14.376	9.177	6.859	5.029	4.814	4.179	3.555	3.262	3.057
Cumulative %	25.129	39.505	48.682	55.541	60.570	65.384	69.563	73.119	76.381	79.437

**Table 2:** The contributions (in percentage) of the variables to the principal components at both locations.

	PC1	PC2	PC3	PC4	PC5
Alanine	6.765	4.495	0.585	1.187	1.068
Sarcosine	10.274	0.019	0.545	0.836	3.513
2-ethylhexanoic acid	0.795	5.703	3.988	0.306	16.973
Pyrrole-2-carboxylic acid	8.908	1.264	0.490	0.035	0.724
Hydroquinone	8.594	1.150	0.000	0.481	0.833
Lactic acid dimer	1.002	0.874	1.002	3.682	1.314
Glutaric acid	7.261	0.779	1.434	10.164	1.215
2-methylmalic acid	3.209	0.204	1.955	14.184	2.735
Decanoic acid	5.810	4.935	0.602	4.949	0.100
Maleamic acid	3.111	1.410	4.615	1.705	5.549
Tropic acid	4.242	0.119	0.194	14.186	0.169
2-methylaminomethyltartronic acid	1.297	3.639	19.472	0.458	1.515
1,4-lactonearabinonic acid	9.316	6.009	1.164	0.587	0.028
trans-1,2-cyclohexanedicarboxylic acid	2.585	4.775	8.674	3.902	0.045
4-hydroxyphenylacetonitrile	6.637	1.940	1.346	0.071	0.213
4-hydroxybenzoic acid	7.872	0.781	1.645	1.925	0.020
Cembrene	8.453	0.113	1.425	2.576	3.984
9-(Z)-hexadecenoic acid	0.367	9.357	18.882	0.370	5.535
Glycyl proline	0.328	6.086	0.070	12.346	2.633
Heptadecanoic acid	0.593	10.605	18.170	0.300	4.567
Sophorose	0.216	16.059	3.712	1.493	1.970
2-deoxyguanosine	0.872	0.002	0.257	17.515	5.524
Secologanin	0.130	1.619	2.947	1.579	14.717
(3α,5β)-3,21-dihydroxypregnane-11,20-dione	0.018	16.913	4.944	0.088	0.048
Varietal resistance to FHB	1.345	1.149	1.883	5.073	25.005

## Data Availability

The data presented in this study are available in the article or supplementary material. The raw MS files are available on request from the corresponding author.

## Supplementary Materials

The following supporting information can be downloaded at: https://www.mdpi.com/article/10.3390/plants12040911/s1, Figure S1: Peak intensities of amino acids and amines (a), benzyl cyanides (b), carbohydrates (c), hydroxysteroids (d), nucleotides (e), polyphenols and their derivatives (f), saturated fatty acids (g), small organic (carboxylic) acids (h), terpenoids (i), and saturated fatty acids (j) in control plants and plants under *Fusarium* treatment at the Osijek experimental location; Figure S2: Peak intensities of amino acids and amines (a), benzyl cyanides (b), carbohydrates (c), hydroxysteroids (d), nucleotides (e), polyphenols and their derivatives (f), saturated fatty acids (g), small organic (carboxylic) acids (h), terpenoids (i), and saturated fatty acids (j) in control plants and plants under *Fusarium* treatment at experimental Tovarnik; Figure S3: Climate diagram for May 2020 at Osijek; Figure S4: Climate diagram for May 2020 at Tovarnik; Table S1: Mann–Whitney U test for 275 metabolites across different treatments at two locations together; Table S2: Area under disease progress curve (AUDPC) for general resistance at experimental locations Osijek and Tovarnik; Table S3: Results for clusters of the agglomerative hierarchical clustering; Table S4: Spearman correlation matrix of 24 wheat metabolites in grains and varietal resistance to *Fusarium*.
