# Supplementary material for: Metabolic Profiling Identifies Changes in the Winter Wheat Grains Following Fusarium Treatment at Two Locations in Croatia
Source: Plants (Basel). 2023 Feb 17;12(4):911. doi: 10.3390/plants12040911 (PMC9962043; doi:10.3390/plants12040911)
Supplement: Supplementary file 1 [file plants-12-00911-s001.zip › plants-2218304 Supplementary/Supplementary Figure S1.docx]

Figure S1. Peak intensities of amino acids and amines (a), benzyl cyanides (b), carbohydrates (c), hydroxysteroids (d), nucleotides (e), polyphenols and their derivatives (f), saturated fatty acids (g), small organic (carboxylic) acids (h), terpenoids (i), and saturated fatty acids (j) in control plants and plants under *Fusarium* treatment at experimental location Osijek.

(a)


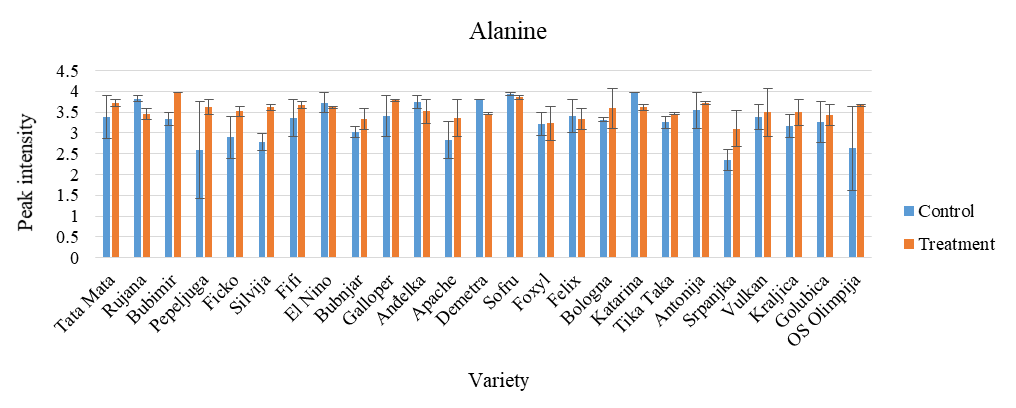

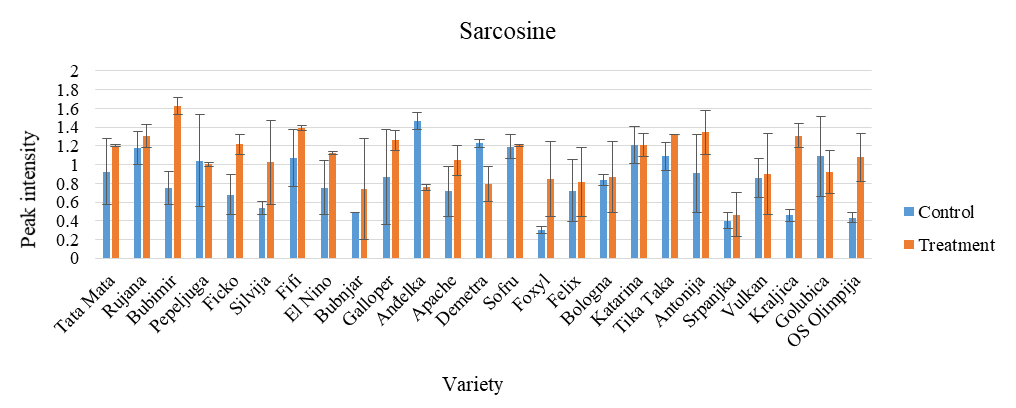

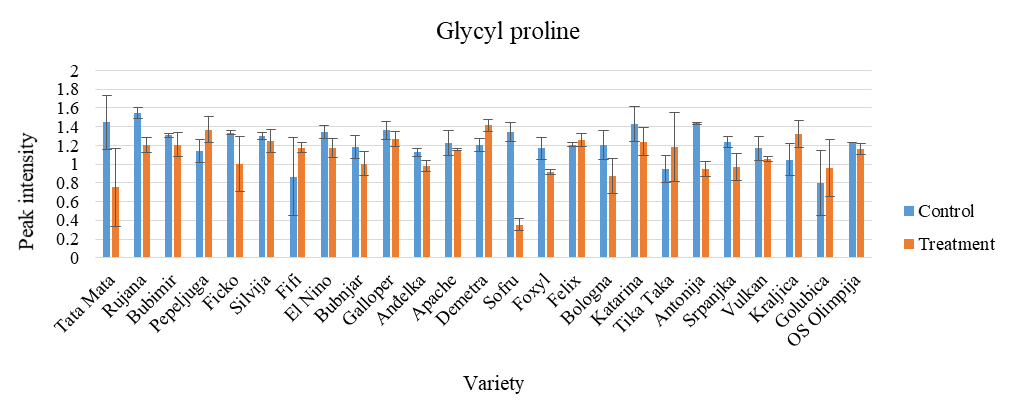


(b) (c)

(d)

(e)

(f)


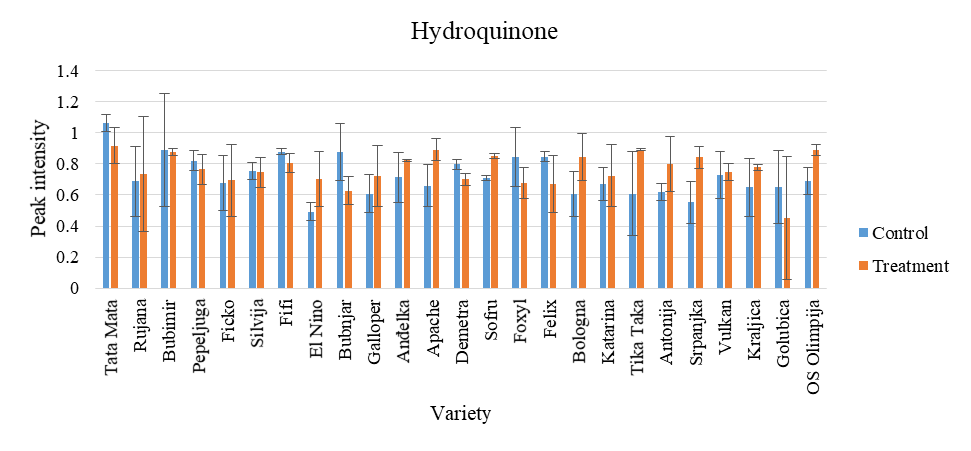

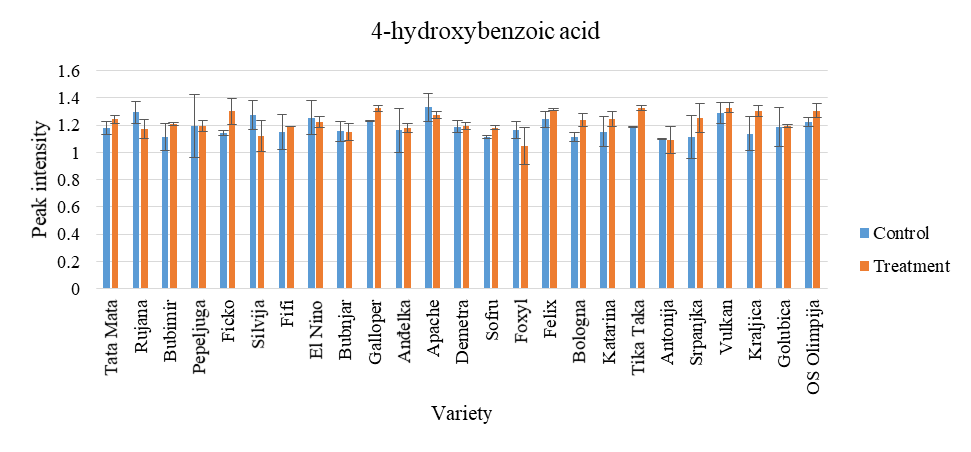


(g)


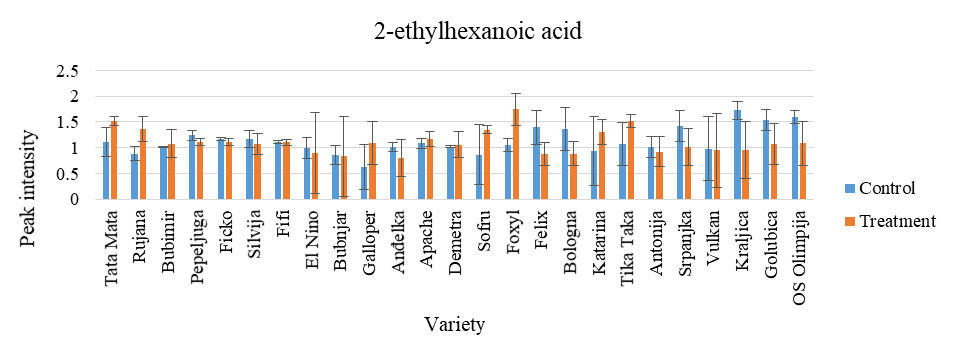

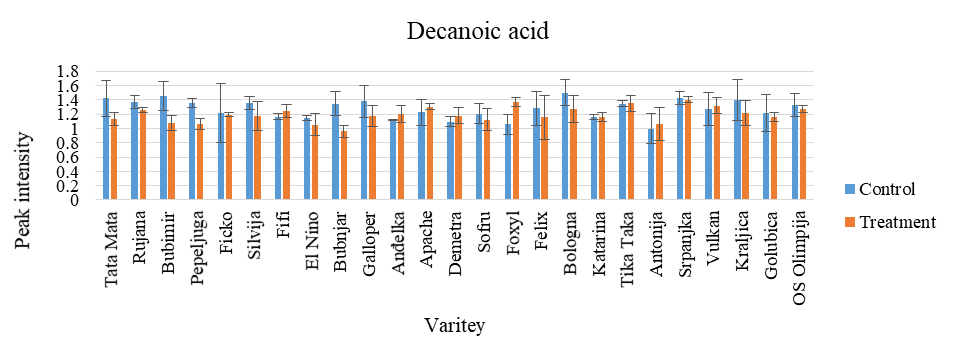

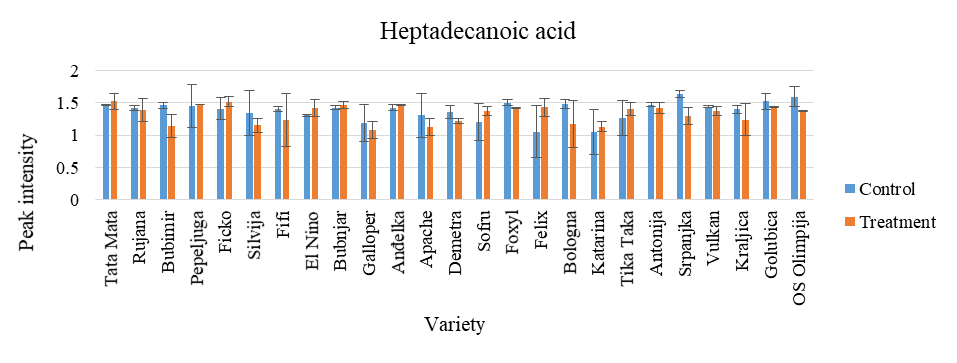


(h)


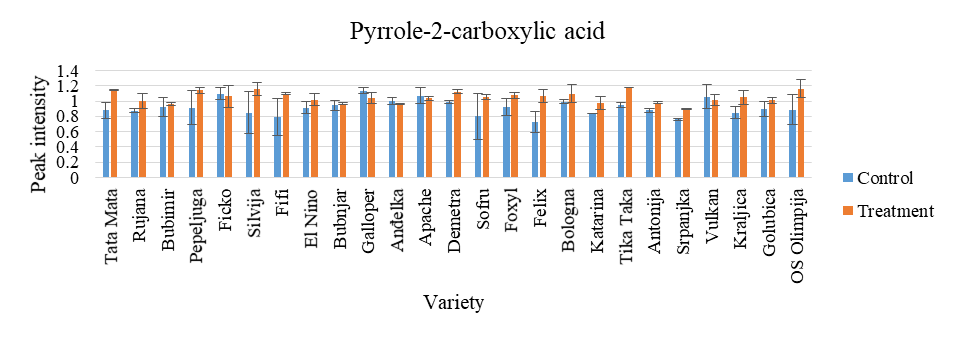

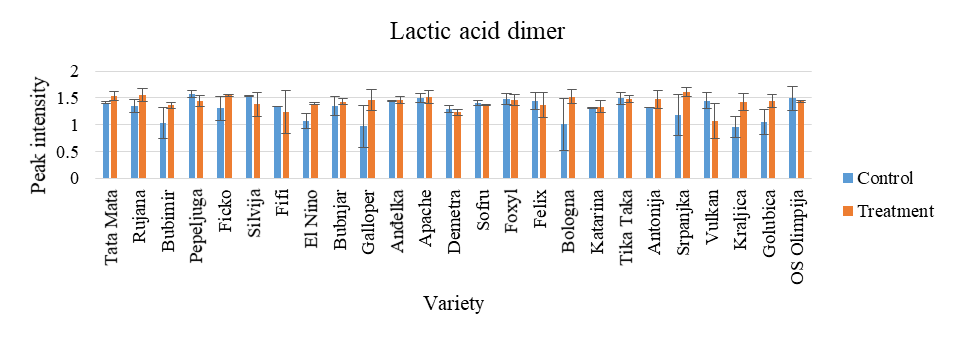

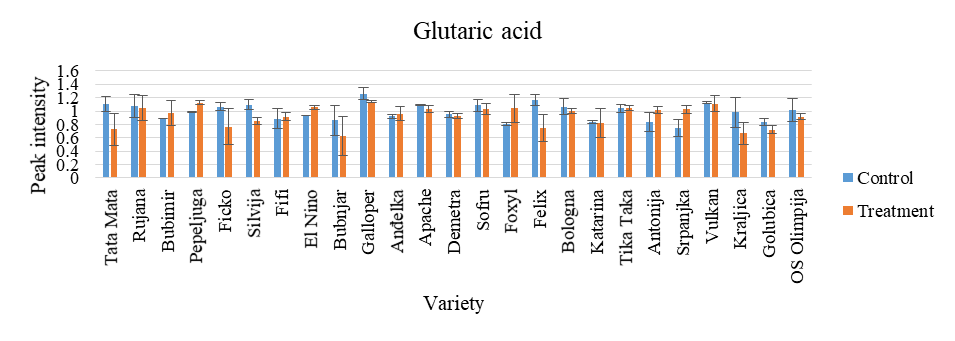

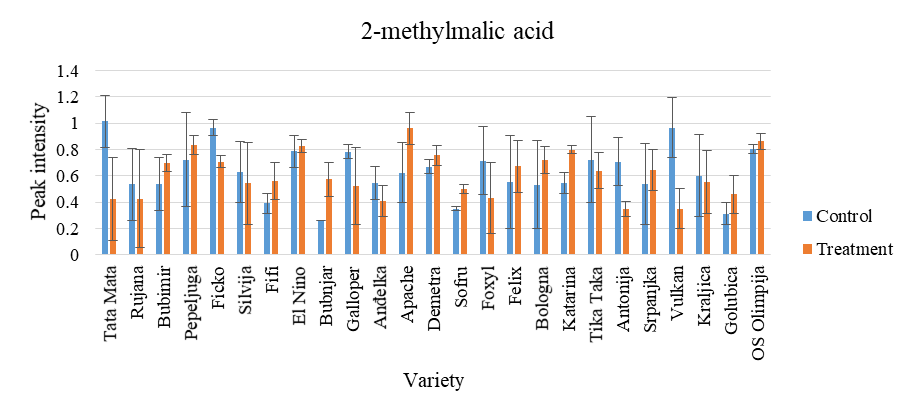

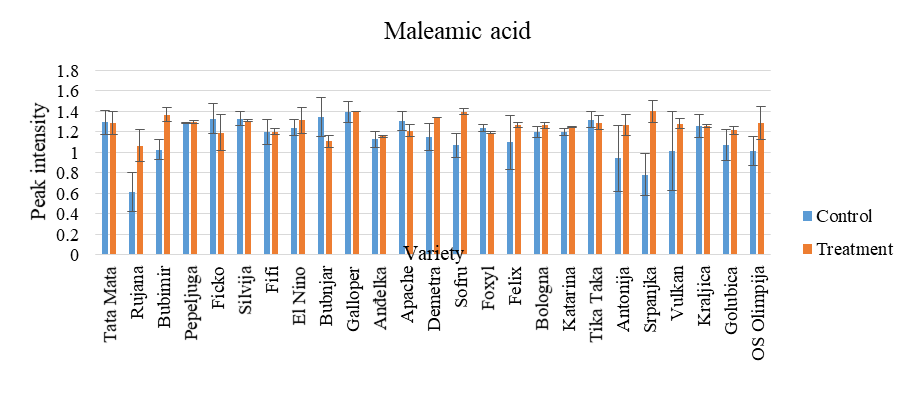

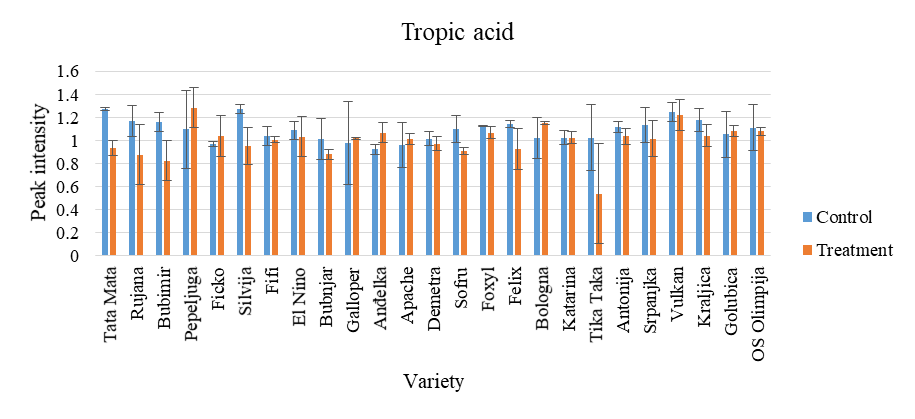

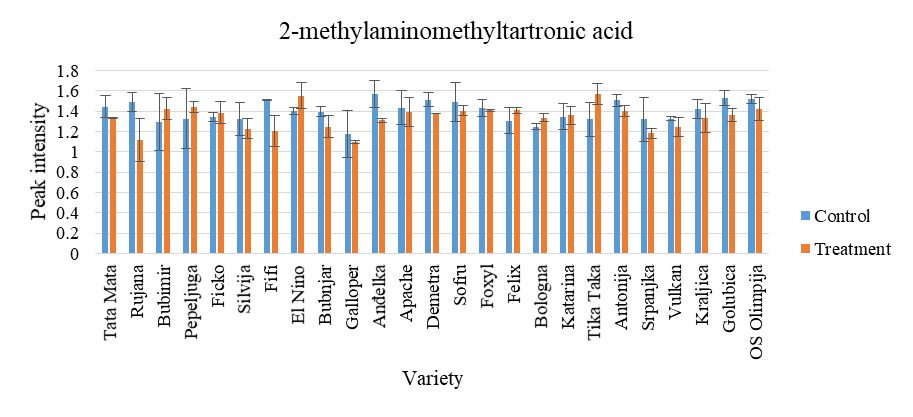

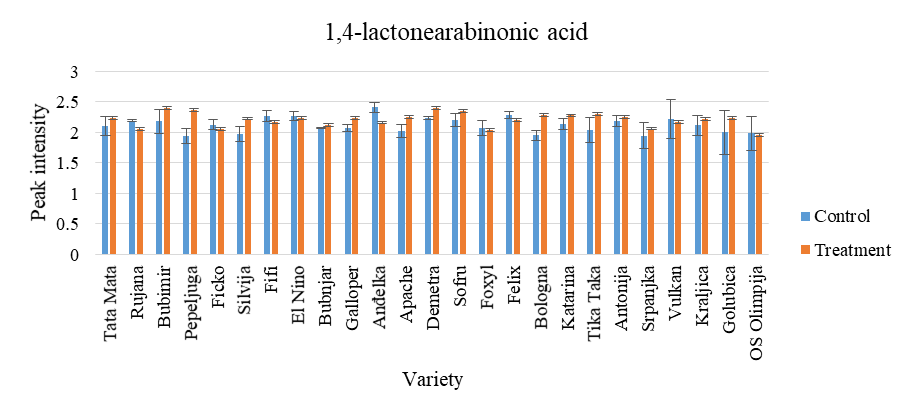

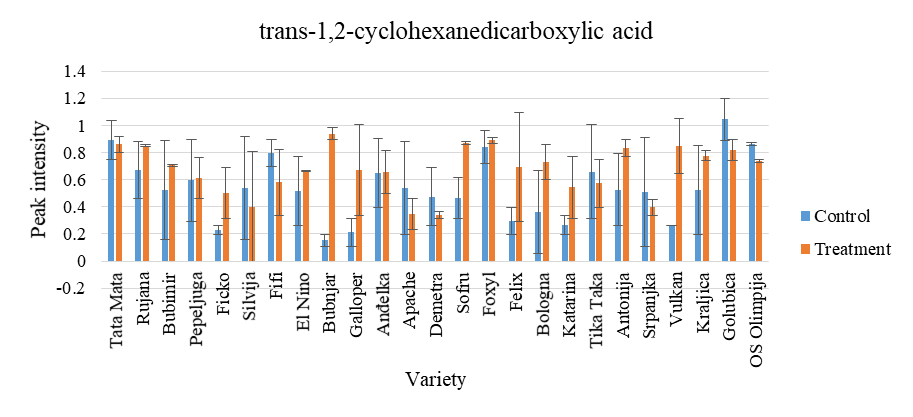


(i)


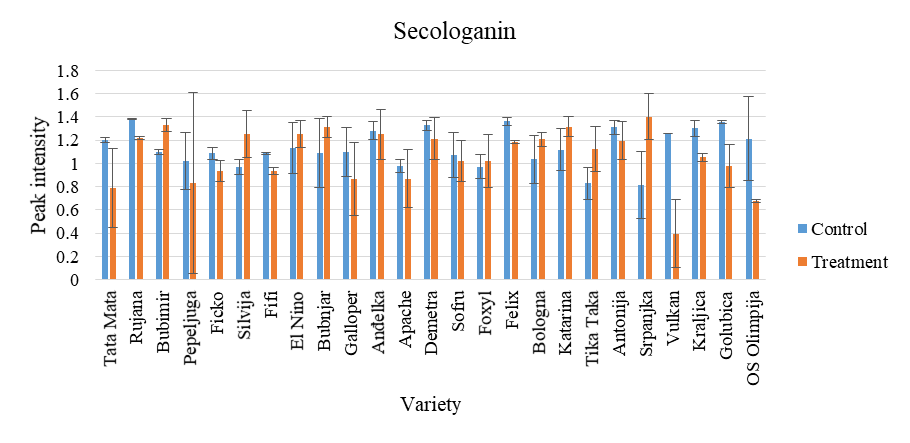

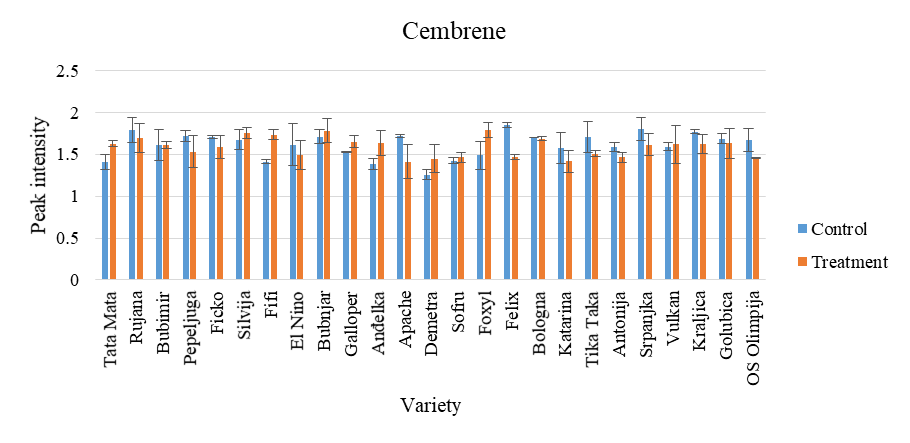


(j)
